# Safe physical interaction with cobots: a multi-modal fusion approach for health monitoring

**DOI:** 10.3389/fnbot.2023.1265936

**Published:** 2023-12-04

**Authors:** Bo Guo, Huaming Liu, Lei Niu

**Affiliations:** ^1^School of Computer and Information Engineering, Fuyang Normal University, Fuyang, China; ^2^Department of Computing, Faculty of Communication, Visual Art and Computing, Universiti Selangor, Selangor, Malaysia

**Keywords:** cognitive workload, biomechanical strain, muscle fatigue, human-machine, interaction cobots

## Abstract

Health monitoring is a critical aspect of personalized healthcare, enabling early detection, and intervention for various medical conditions. The emergence of cloud-based robot-assisted systems has opened new possibilities for efficient and remote health monitoring. In this paper, we present a Transformer-based Multi-modal Fusion approach for health monitoring, focusing on the effects of cognitive workload, assessment of cognitive workload in human-machine collaboration, and acceptability in human-machine interactions. Additionally, we investigate biomechanical strain measurement and evaluation, utilizing wearable devices to assess biomechanical risks in working environments. Furthermore, we study muscle fatigue assessment during collaborative tasks and propose methods for improving safe physical interaction with cobots. Our approach integrates multi-modal data, including visual, audio, and sensor- based inputs, enabling a holistic assessment of an individual's health status. The core of our method lies in leveraging the powerful Transformer model, known for its ability to capture complex relationships in sequential data. Through effective fusion and representation learning, our approach extracts meaningful features for accurate health monitoring. Experimental results on diverse datasets demonstrate the superiority of our Transformer-based multi- modal fusion approach, outperforming existing methods in capturing intricate patterns and predicting health conditions. The significance of our research lies in revolutionizing remote health monitoring, providing more accurate, and personalized healthcare services.

## 1 Introduction

Health monitoring is a crucial aspect of modern healthcare systems, allowing for the early detection and management of various medical conditions. Traditional approaches to health monitoring often rely on single-mode data, such as medical records or sensor readings, which provide limited insight into an individual's overall health status. In recent years, the advent of cloud-based robot-assisted systems has offered promising opportunities for more efficient and personalized health monitoring. Cloud-based robot-assisted systems combine the power of cloud computing with the capabilities of robotic devices to enable remote monitoring and intervention. These systems have the potential to revolutionize healthcare by providing continuous, real-time monitoring of vital signs, activity levels, and other relevant health indicators. This allows for proactive healthcare interventions, timely disease management, and improved overall well-being. One of the key challenges in health monitoring within cloud-based robot-assisted systems is the effective integration and analysis of multi-modal data. Multi-modal data refers to information collected from various sources, such as visual data from cameras, audio data from microphones, and sensor data from wearable devices. Integrating and fusing such diverse data types can provide a more comprehensive and accurate understanding of an individual's health status. To address this challenge, various models have been proposed in the field of multi-modal health monitoring. Here, we introduce five relevant models:

Convolutional Neural Networks (CNNs): CNNs have been widely used for analyzing visual data in health monitoring systems. They excel at extracting spatial features and have been applied to tasks such as facial expression recognition and activity recognition.

Recurrent Neural Networks (RNNs): RNNs are effective in capturing temporal dependencies in sequential data. They have been employed in health monitoring applications for analyzing time-series sensor data, such as heart rate and accelerometer readings.

Graph Neural Networks (GNNs): GNNs are designed to model relationships and interactions among entities in graph-structured data. In health monitoring, GNNs can capture correlations between different health indicators, such as the relationships between blood pressure and heart rate.

Generative Adversarial Networks (GANs): GANs are commonly used for generating synthetic data that closely resemble real data. In health monitoring, GANs can be utilized to augment the limited training data and enhance the diversity of the dataset.

Transformer Models: Transformers have gained significant attention in natural language processing tasks but have also shown promise in handling sequential and structured data. In health monitoring, Transformer models can capture complex relationships among multi-modal data sources, enabling effective fusion, and representation learning.

The objective of this research is to propose a Transformer-based Multi-modal Fusion approach for health monitoring in cloud-based robot-assisted systems. By leveraging the strengths of the aforementioned models, we aim to provide a comprehensive and accurate assessment of individuals' health status, leading to improved diagnosis, intervention, and overall healthcare outcomes. This paper makes the following three main contributions to the field of health monitoring in cloud-based robot-assisted systems:

1. We propose a Transformer-based Multi-modal Fusion approach that leverages the power of Transformers to effectively integrate and fuse multi-modal data. By applying the Transformer architecture, our method captures complex relationships and dependencies among different data sources, leading to a more comprehensive and accurate assessment of an individual's health status.
2. Our approach addresses the limitations of traditional unimodal methods by utilizing multi-modal data, including visual, audio, and sensor-based inputs. This allows for a more holistic understanding of an individual's health condition, enabling early detection, and intervention for various medical conditions.
3. We conducted extensive experiments to evaluate the performance of our proposed method. By comparing it with existing approaches, we demonstrate the superiority of our Transformer-based Multi-modal Fusion approach in capturing intricate patterns and accurately predicting health conditions. These results provide empirical evidence of the effectiveness and potential impact of our approach in improving health monitoring within cloud-based robot-assisted systems.

## 2 Related work

### 2.1 Health monitoring in cloud-based robot-assisted systems

The advancements in robotics technology have significantly impacted various aspects of society, the economy, and people's daily lives. With the emergence of wireless network technology and cloud computing, robots have transitioned from the industrial control field to the service field (Turnbull and Samanta, 2013). In the current market, robots primarily focus on family education, entertainment, and domestic services, such as cleaning robots. However, these robots are often limited by their standalone functionality, lack of intelligence, and difficulties in maintenance and upgrades. To overcome these limitations, the concept of networked robots has been introduced, enabling remote operation and management, as well as multi-robot cooperation. This has led to the development of cloud robots (Zheng et al., 2012; Kehoe et al., 2015). The architecture of cloud robots consists of two tiers: the machine-to-machine (M2M) level (Chen, 2013) and the machine-to-cloud (M2C) level (Hu et al., 2012). At the M2M level, a group of robots is connected through wireless networks to form an *ad-hoc* robot collaborative cloud infrastructure. The M2C level provides a shared computing and storage resource pool, allowing robots to offload computing tasks to the cloud. The networked robots in the M2M level can communicate and collaborate with each other, enabling complex tasks to be accomplished through distributed computation and cooperation. The M2C level provides additional capabilities by leveraging the power of cloud computing and storage resources. This allows robots to access advanced data processing algorithms, machine learning models, and large-scale data storage to enhance their intelligence and expand their capabilities (Wang et al., 2022).

By integrating wireless networks and cloud computing, cloud robots can achieve enhanced performance, scalability, and intelligence. They can leverage powerful computing resources, access vast amounts of data, and benefit from shared intelligence and learning. This enables them to perform more complex tasks, adapt to changing environments, and continuously improve over time (Tian et al., 2023).

### 2.2 Multi-modal robotics

The existing approaches for visual language representation predominantly rely on BERT-style training objectives to capture cross-modal alignments (Devlin et al., 2018). These approaches have been widely applied in various downstream tasks, including visual question answering, grounding, retrieval, and captioning (Lu et al., 2019; Sun et al., 2019; Zhou et al., 2020). However, learning representations for robotics tasks presents additional challenges due to the conditioning of perception data on motion policies and model dynamics (Shi et al., 2023b).

Visual-language navigation of embodied agents is a well-established field within robotics, featuring established benchmarks and simulators (Anderson et al., 2018; Shi et al., 2023a). In recent years, several studies have focused on exploring the alignment of vision and language data for robotics applications. These studies have typically involved combining pretrained models with fine-tuning techniques (Nguyen and Daumé, 2019; Hao et al., 2020; Thomason et al., 2020). To further enhance the modeling of visual-language alignment in robotics, researchers have proposed innovative approaches. One such approach is the co-grounding attention mechanism, which aims to dynamically align visual and language information based on shared semantic understanding (Ma et al., 2019). By leveraging this mechanism, models can effectively capture cross-modal correlations and improve their overall understanding of the environment. In the domain of manipulation, there has been notable work by Zhao and Lv (2023), which utilizes CLIP (Radford et al., 2021) embeddings to integrate semantic and spatial information. By leveraging the embedding space provided by CLIP, the manipulation model gains the capability to reason about objects' semantics and their spatial relationships. This integration allows for more effective planning and execution of manipulation tasks within a robotic system.

### 2.3 Transformer-based models in robotics

Transformers have become a prevalent architectural choice in various domains, making significant contributions to NLP (Brown et al., 2020; Shi et al., 2023b), vision (Dosovitskiy et al., 2020; Liu et al., 2021), and even reinforcement learning (Chen et al., 2021; Lee et al., 2022). Additionally, transformers have found applications in the field of robotics, demonstrating their versatility, and effectiveness in addressing a wide range of robotic tasks.

In the realm of robotics, transformers have been successfully applied to assistive teleoperation (Janner et al., 2021), legged locomotion (Yang et al., 2021), path planning (Chaplot et al., 2021), imitation learning (Kim et al., 2021), morphology controllers (Gupta et al., 2022), spatial rearrangement (Liu et al., 2022), and grasping (Han et al., 2021). These applications highlight the capability of transformers in addressing diverse robotic challenges across different domains. For instance, in assistive teleoperation, transformers can assist human operators by leveraging their ability to model long-range dependencies and capture complex spatial relationships. In legged locomotion, transformers enable robots to analyze and predict motion patterns, facilitating agile and dynamic movements. Furthermore, transformers have shown impressive results in multi-domain settings. Projects like Gato (Reed et al., 2022) have trained a single transformer on 16 domains, including captioning, language grounding, and robotic control. Despite the magnitude of the task, Gato achieved remarkable performance. However, it is worth mentioning that some of these achievements rely on large amounts of data, such as 15,000 episodes for block stacking and 94,000 episodes for Meta-World tasks (Yu et al., 2020). These extensive datasets are crucial for learning intricate representations and achieving high-level performance in complex robotic scenarios.

While these studies demonstrate the potential of Transformer-based models in health monitoring, there is still a need for research specifically focused on the fusion of multi-modal data using Transformers in cloud-based robot-assisted systems. In this paper, we build upon the existing literature by proposing a Transformer-based Multi-modal Fusion approach tailored for health monitoring in cloud-based robot-assisted systems. Our approach aims to leverage the strengths of Transformers in capturing complex relationships and dependencies among multi-modal data sources, providing a comprehensive and accurate assessment of an individual's health status.

## 3 Method

**Figure 1:**
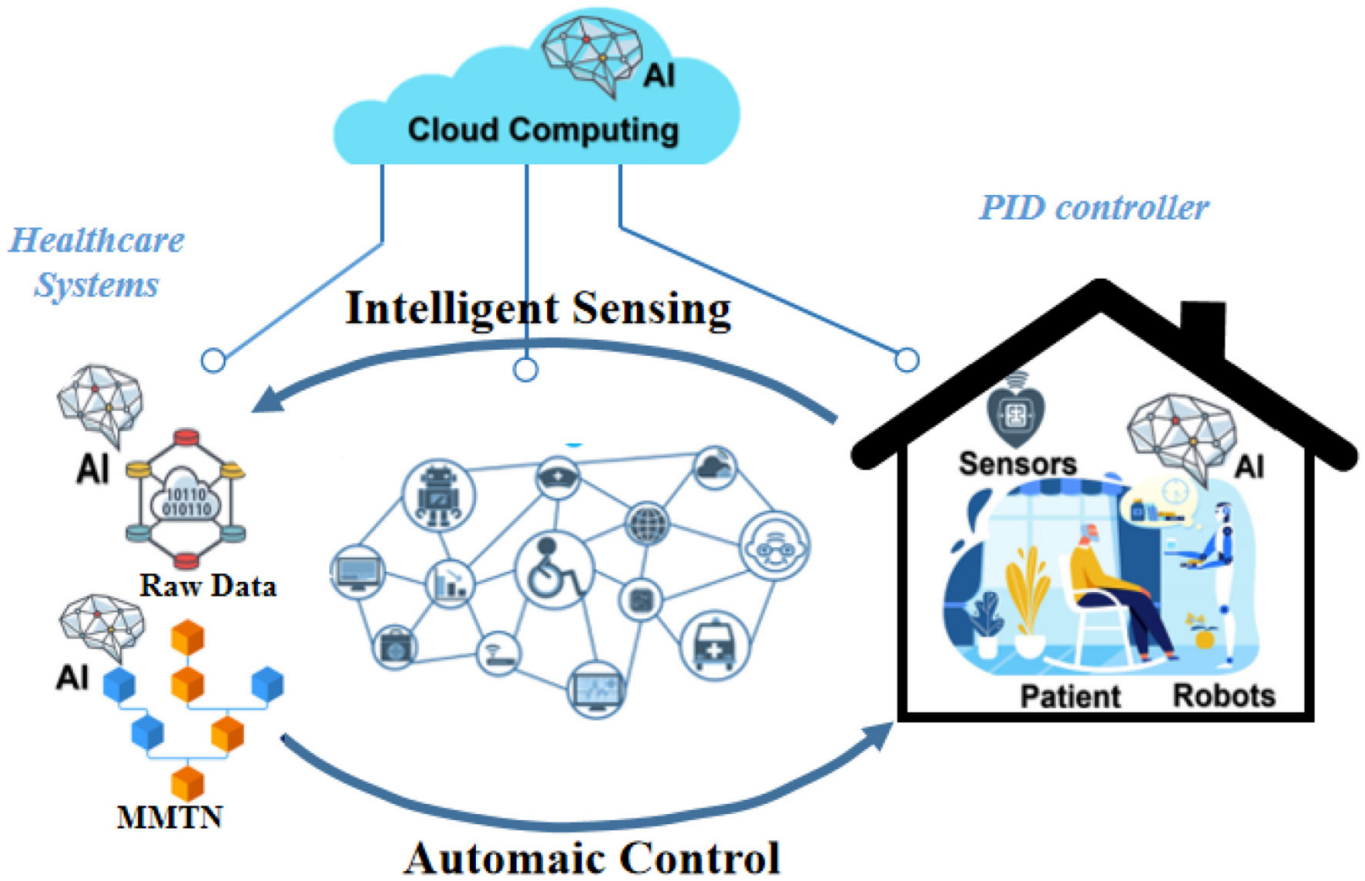
Workflow of transformer-based multi-modal fusion for health monitoring in cloud-based robot-assisted systems.

**Figure 2:**
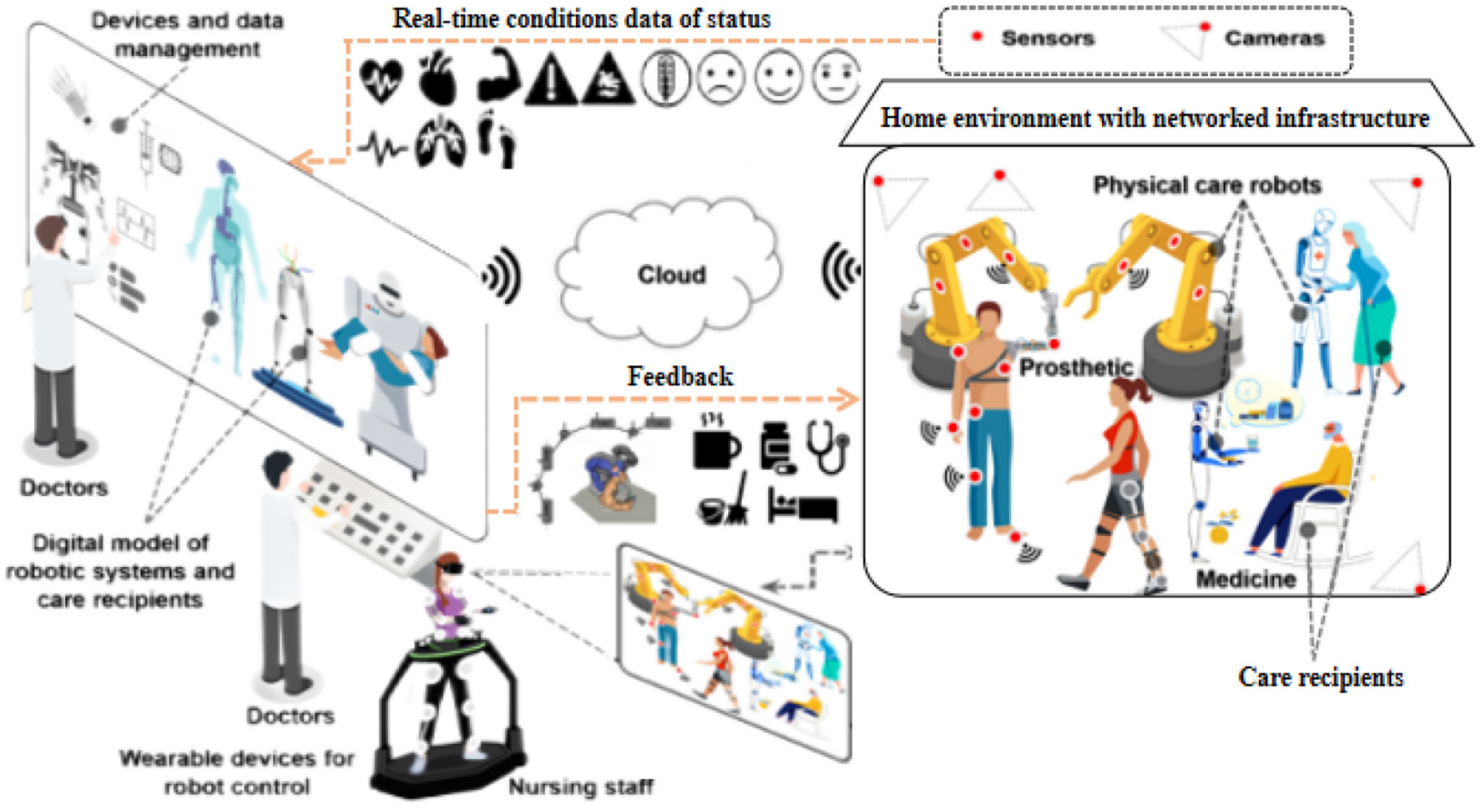
Workflow of transformer-based multi-modal fusion for health monitoring in cloud-based robot-assisted systems.

In this section, we present our proposed method, Transformer-based Multi-modal Fusion for Health Monitoring in Cloud-based Robot-assisted Systems. The overall workflow of our approach is illustrated in Figures 1, 2.

**Figure 3:**
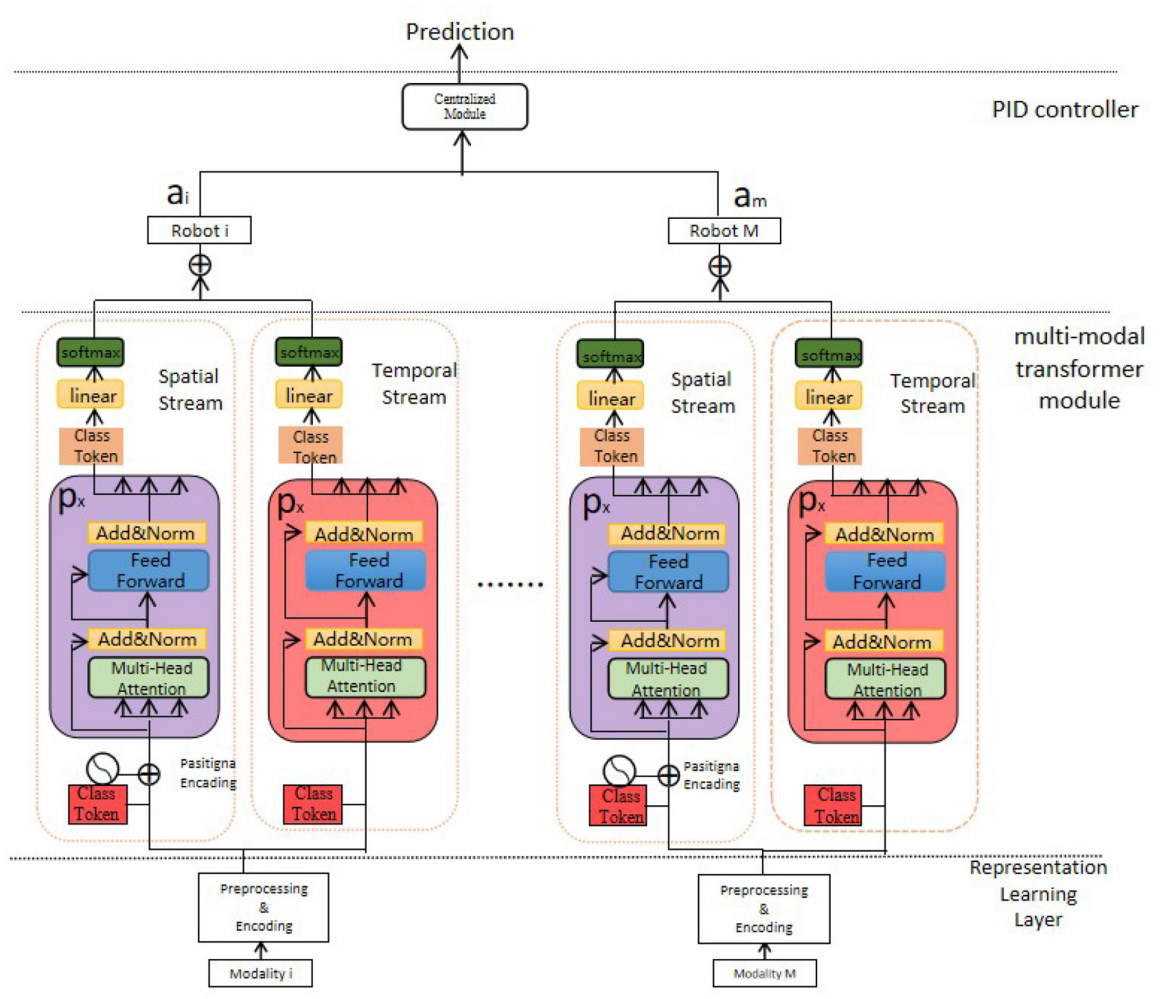
The multi-modal transformer networks.

Our MMTN (Multi-modal Transformer Networks) model is shown in Figure 3, which consists of three key components:

Generalized Representation Learning Layer: This layer receives the raw data collected from various sensors embedded in the robot and conducts necessary preprocessing steps for further feature extraction. It ensures that the data is in a suitable format and ready for subsequent processing.

Multi-modal Transformer Module: This module is responsible for extracting salient features from each modality within the multi-modal data. By utilizing transformer architecture, it captures the interdependencies among different modalities effectively. Additionally, it generates the representation of class tokens, which encodes comprehensive information regarding the health status of the robot.

Proportional-Integral-Derivative (PID) Controller: In our method, we employ a PID controller to guide the actions of the robot in cloud-based robot-assisted systems. The controller utilizes the comprehensive health status representation obtained from the multi-modal transformer module to determine potential risks, symptom developments, and overall health state of the robot. It enables timely and appropriate responses to ensure the safety and efficiency of the system.

The pseudocode outlines the training process of a deep learning model that integrates Multi-modal Fusion, Transformer, and PID control strategies. The model takes four different training datasets: “MIMIC-III,” “PhysioNet,” “UCI Machine Learning Repository,” and “Open-i.” It starts by initializing the Multi-modal Fusion model, Transformer model, and PID gains. The learning rate and the number of epochs are then set. For each epoch, the model performs multi-modal fusion, encodes the fused features using a Transformer, applies multi-head self-attention, and uses a feed-forward neural network. A PID control signal is computed and used to update the model parameters using gradient descent. This process is repeated until convergence criteria are met. If the validation loss decreases, the best model is saved. The final output is the trained model parameters.

**Algorithm 1:**
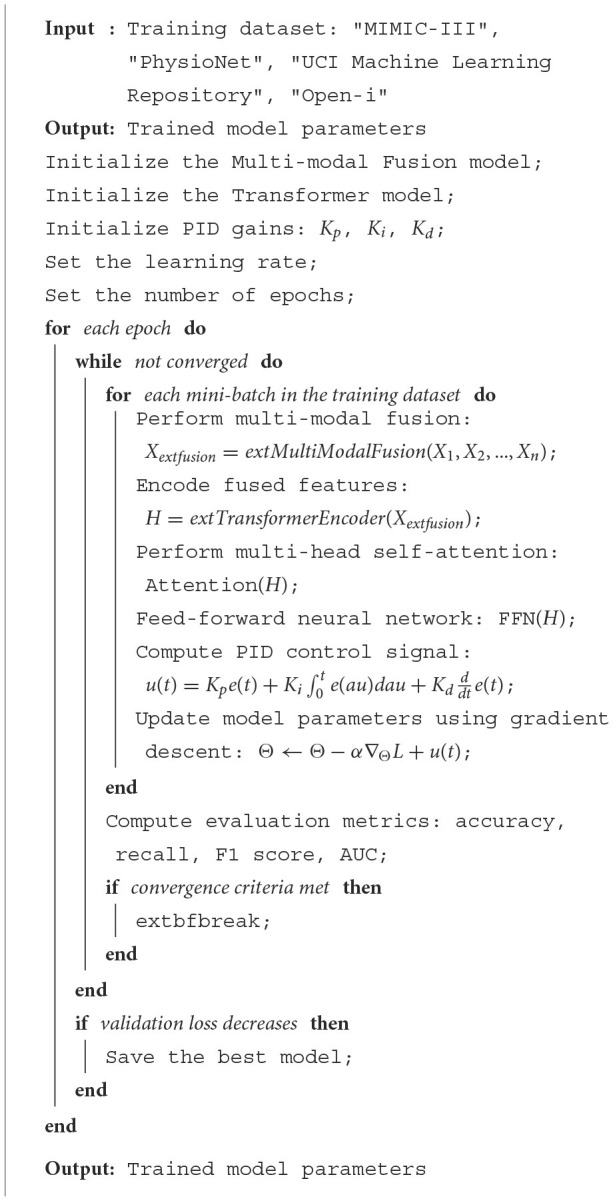
Multi-modal fusion, transformer, and PID training.

### 3.1 Unified representation learning

MMTN first takes the raw multimodal data and transfers each modality into a unified representation to be fed into the MSTT module.

The dataset D is a set of data records of all the modalities, where $d_{m}^{n}$ is the *n*th record of modality m.


(1) $$\begin{array}{l} D=\left[\begin{array}{ccc} d_{1}^{1} & \cdots & d_{M}^{1}\\ \vdots & \ddots & \vdots \\ d_{1}^{N} & \cdots & d_{M}^{N} \end{array}\right] \end{array}$$

For record *n*, denotes the record at timestep t. It is worth noting $d_{m,t}^{n}$ that could either be a one-dimensional vector (sensor or skeleton data) or a two-dimensional matrix (visual data, excluding the channel dimension) depending on the input data format.

Our method has two advantages. First, we use a common method to generate unified representations for all the modalities, which can be easily generalized to a new modality. This would not require complex encoder architecture and extra data engineering. Second, our approach could utilize a pre-trained model and be completed offline. As it does not require a sequential Neural Networks module, the computation can be done in parallel and easily scale when a new modality is introduced, which improves the computation cost during both training and testing.

### 3.2 Multi-modal transformer model

The Transformer model serves as the core component of our approach. It is a deep learning architecture that employs self-attention mechanisms to capture the relationships between different elements in a sequence. In our case, the input sequence consists of visual and textual information extracted from various sensors. The Transformer model consists of two key modules: the Encoder and the Decoder. The Encoder module takes the input sequence and applies self-attention mechanisms to capture the dependencies between different modalities. It produces a modality-specific representation for each input modality, considering both the local and global contexts. The Decoder module predicts the health condition or detects anomalies based on the fused representations from the Encoder module. In this multi-modal fusion approach, the Transformer model plays a pivotal role in integrating the visual and textual information. It learns to assign appropriate weights to each modality, considering their relevance and contribution to the overall prediction. By dynamically attending to different modalities, the Transformer model effectively fuses the information and generates a comprehensive representation. The Transformer-based Multi-modal Fusion approach harnesses the power of the Transformer model to improve health monitoring in cloud-based robot-assisted systems. By utilizing the self-attention mechanisms in the Transformer, our approach captures intricate relationships between visual and textual cues, enabling a more holistic understanding of the health conditions. The Transformer model not only handles the fusion of multi-modal information but also captures contextual dependencies within each modality. This enables our approach to adaptively weigh the importance of each modality during the fusion process, leading to more accurate predictions and anomaly detection.

The Transformer model used in our proposed approach is based on a series of mathematical equations that govern the flow of information within the model. These equations involve several variables, each with its own specific interpretation. Here, we provide an overview of the main equations along with the explanation of the variables involved: 1. Self-Attention Mechanism: The self-attention mechanism allows the Transformer model to capture the dependencies between different elements within a sequence. It calculates attention weights for each element based on its relationship with other elements in the sequence. 2. Given an input sequence X, the self-attention mechanism calculates the attention weights using the following equations:


(2) $$\begin{array}{l} Attention\left(Q,K,V\right)=softmax\left(\frac{QK^{T}}{\sqrt{d_{k}}}\right)V \end{array}$$

Here, Q, K, and V represent query, key, and value matrices, respectively. *d*_*k*_ denotes the dimension of the key vectors. The softmax function normalizes the logits, producing attention weights that sum up to 1.

3. Encoder-Decoder Attention: The encoder-decoder attention is responsible for capturing the dependencies between the encoder and decoder representations in the Transformer model. It aligns the decoder's current position with the relevant encoder positions. This attention mechanism is crucial for generating context-aware representations in the decoder.

Similar to the self-attention mechanism, the encoder-decoder attention is calculated using the following equations:


(3) $$\begin{array}{l} Attention\left(Q,K,V\right)=softmax\left(\frac{QK^{T}}{\sqrt{d_{k}}}\right)V \end{array}$$

4. Position-wise Feed-Forward Networks: The position-wise feed-forward networks consist of two fully connected layers with a ReLU activation function applied in between. These networks serve as non-linear transformations applied independently to each position in the sequence.

The output of the position-wise feed-forward networks can be expressed as:


(4) $$\begin{array}{l} FFN\left(x\right)=max\left(0,xW_{1}+b_{1}\right)W_{2}+b_{2} \end{array}$$

Here, x denotes the input vector representations, *W*_1_, *W*_2_, *b*_1_, and *b*_2_ represent the weight matrices and bias terms, respectively. These equations collectively define the computations involved in the Transformer model, facilitating multi-modal fusion, and enhanced health monitoring in cloud-based robot-assisted systems.

### 3.3 Robot-assisted systems

**Figure 4:**
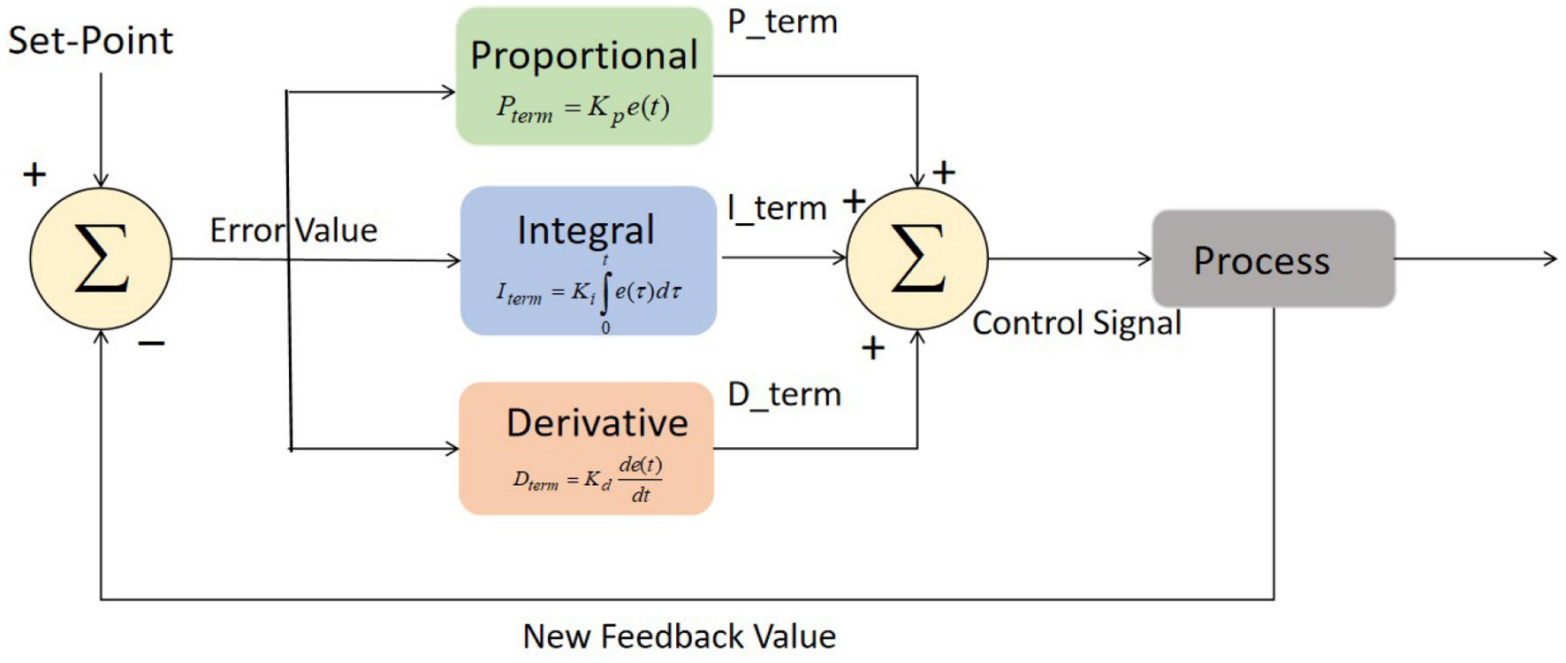
Block diagram of PID controller.

The control algorithm used in this study incorporates a Proportional-Integral-Derivative (PID) controller for controlling the actions of the robot in cloud-based robot-assisted systems. The PID controller is a widely used feedback control mechanism that adjusts the control signal based on the error between the desired state and the current state of the system. The basic structure of PID controller is depicted in Figure 4. The main components of PID controller are optimization techniques, process, fitness function, and sensors.

The PID control algorithm can be described as follows:


(5) $$u(t)=K_{p}\cdot e(t)+K_{i}\cdot \int_{0}^{t}e(\tau )d\tau +K_{d}\cdot \frac{de(t)}{dt}$$

where:

*u*(*t*) represents the control signal generated by the PID controller, which determines the robot's actions. *K*_*p*_, *K*_*i*_, and *K*_*d*_ are the proportional, integral, and derivative gains, respectively. These gains are determined through tuning and affect the contribution of each term to the control signal. *e*(*t*) represents the error at time *t*, which is calculated as the difference between the desired state and the current state of the system. The integral term, given by $K_{i}\cdot \int_{0}^{t}e(\tau )d\tau$, accumulates the error over time. It accounts for any steady-state errors and adjusts the control signal to reduce them. The derivative term, $K_{d}\cdot \frac{de\left(t\right)}{dt}$, considers the rate of change of the error. It helps in stabilizing the system and damping any oscillations by adjusting the control signal based on the error's rate of change. The PID gains *K*_*p*_, *K*_*i*_, and *K*_*d*_ are typically determined through a process called tuning, where they are adjusted iteratively to achieve the desired system response. Systematic tuning methods such as Ziegler-Nichols or Cohen-Coon methods can be employed to determine the optimal values of these gains for achieving stability, responsiveness, and accuracy in controlling the robot's actions.

By utilizing the PID control algorithm, the control algorithm guides the robot's actions in real-time based on the error between the desired state and the current state of the system. This enables the robot to respond dynamically to changes in the environment or user's condition, providing precise and reliable assistance in cloud-based robot-assisted systems.

## 4 Experiments

### 4.1 Datasets

For evaluating the proposed Transformer-based Multi-modal Fusion approach in cloud-based robot-assisted health monitoring, we utilize four publicly available datasets that encompass diverse clinical data and facilitate comprehensive analysis shown in Table 1.

**Table 1:** Overview of the datasets used in the study.

**Dataset**	**Key features**	**Number of instances**
MIMIC-III	EHRs, physiological waveforms, lab measurements, medications	46,520 patients
PhysioNet	ECG, EEG, blood pressure recordings	70 recordings
UCI	Disease diagnosis, medical imaging, patient monitoring	31 people
Open-i	Radiological images, clinical photographs, microscopy images	3,996 reports

MIMIC-III (Medical Information Mart for Intensive Care III; Johnson et al., 2016): We leverage the MIMIC-III dataset, a widely used resource containing comprehensive clinical data from ICUs in a large academic medical center. This dataset includes electronic health records, physiological waveforms, laboratory measurements, medications, and clinical notes. Its extensive nature allows for a holistic assessment of patients' health conditions in an ICU setting. The MIMIC-III database contains 46,520 patients at the unique patient level, of whom 38,163 are adult patients, 38,161 patients were last admitted to the ICU, and 38,156 patients have the first diagnosis.

PhysioNet (Moody et al., 2001): The PhysioNet repository provides access to a variety of physiological signal datasets, including electrocardiography (ECG), electroencephalography (EEG), and blood pressure recordings. These datasets offer valuable resources for studying the fusion of physiological signals with other modalities, enhancing health monitoring capabilities. PhysioNet Apnea-ECG dataset provided by Philipps University contains a total of 70 single-lead ECG signal recordings (released set: 35 recordings, withheld set: 35 recordings), which were sampled at 100 Hz and ranged between 401 and 587 min. For each 1 min ECG signal recording segment, the dataset provided an expert annotation.

UCI Machine Learning Repository: The UCI Machine Learning RepositoryOpen-i (Little et al., 2007) hosts a diverse collection of healthcare-related datasets, covering areas such as disease diagnosis, medical imaging, and patient monitoring. These datasets serve as valuable sources for developing and evaluating health monitoring algorithms, allowing for controlled experimental settings. The dataset range of biomedical voice measurements from 31 people, where 23 people are showing Parkinson's disease.

Open-i: Open-i (Demner-Fushman et al., 2016) is a publicly available chest X-ray dataset collected by Indiana University. Unlike MIMIC-CXR, which is annotated by auto-annotators, Open-i dataset is labeled by medical professionals manually using Medical Subject Heading (MeSH) indexing. The dataset contains 3,996 radiology reports associated with 8,121 images. Each pair is assigned multiple MeSH terms by human-annotators.

### 4.2 Experimental settings

Before training our model, we performed necessary preprocessing steps on the collected data. This involved resizing the visual data to a consistent size, normalizing pixel values, and augmenting the dataset using techniques such as random flips and rotations to improve training performance. The auditory data underwent preprocessing steps including filtering, noise removal, and feature extraction using Mel Frequency Cepstral Coefficients (MFCCs). The tactile data was preprocessed by removing noise, normalizing values, and converting them into appropriate representations.

For the model configuration, we adjusted key hyperparameters of the Transformer-based multi-modal fusion model. This included determining the number of transformer layers, attention heads, and hidden dimensions based on empirical analysis and model performance. We utilized pre-trained models, such as ResNet and MobileNet, for extracting modality-specific features. The fusion process incorporated attention mechanisms and concatenation layers to effectively combine the multi-modal representations.

In terms of implementation, we used the PyTorch framework to develop and train our model. The experiments were conducted on an NVIDIA Titan RTX GPU to leverage its computational power and speed up the training process. We employed a training-validation-test split for evaluating the model's performance, ensuring that the test set was independent and representative of real-world scenarios.

To evaluate the model, we conducted several experiments, including metric comparison and ablation studies. The metrics considered for comparison included training time (in seconds), inference time (in milliseconds), number of parameters (in millions), and the number of floating-point operations (in billions). Additionally, we evaluated the model's performance using metrics such as accuracy, area under the curve (AUC), recall, and F1 score.

During training, we employed standard techniques such as mini-batch stochastic gradient descent (SGD) with a suitable learning rate schedule and weight decay. We monitored the model's performance on the validation set and employed early stopping to prevent overfitting. Hyperparameters, including learning rate, batch size, and regularization parameters, were tuned through a systematic grid search or Bayesian optimization.

Throughout the experimental process, we carefully documented all hyperparameters, preprocessing steps, and training details to ensure reproducibility. The code implementation and algorithms followed best practices and guidelines in the field.

The training process of the MMTN involves several steps:

Data Preprocessing: Prior to training, the raw multimodal data is preprocessed to ensure compatibility and consistency across different modalities. This preprocessing step may include resizing and normalizing images, converting audio signals to spectrograms, and scaling sensor-based inputs. The aim is to prepare the data for effective training and fusion within the MMTN.

Feature Extraction: Once the data is preprocessed, feature extraction is performed to capture meaningful representations from each modality. This step involves applying appropriate techniques such as convolutional neural networks (CNNs) for visual data, recurrent neural networks (RNNs) for sequential data, or other specialized models for different modalities. The extracted features from each modality are then combined to form a multimodal representation.

Transformer-based Fusion: The MMTN employs a Transformer architecture to fuse the multimodal representations. The Transformer model is composed of multiple layers of self-attention and feed-forward neural networks. During training, the model learns to attend to relevant features from different modalities and capture complex relationships between them. This fusion process helps the MMTN leverage the complementary information present in the multimodal data.

Loss Function and Optimization: To train the MMTN, a suitable loss function is defined based on the specific health monitoring task. This could be a classification loss for predicting health conditions or a regression loss for estimating continuous variables. The model is optimized by minimizing this loss function using gradient-based optimization algorithms such as stochastic gradient descent (SGD) or its variants. Regularization techniques like dropout or weight decay may also be applied to prevent overfitting during training.

Iterative Training: The training process is typically performed iteratively over a large labeled dataset. The MMTN is presented with batches of preprocessed multimodal data, and forward and backward propagation are performed to compute the gradients and update the model parameters. This iterative process continues until the model converges or a predefined stopping criterion is met.

Here are the formulas for each metric along with the explanations of the variables:

Training Time (*T*)—The total time taken for model training in seconds.


(6) $$\begin{array}{l} T \end{array}$$

Inference Time (*I*)—The average time taken for the model to make predictions on a single sample in milliseconds.


(7) $$\begin{array}{l} I \end{array}$$

Parameters (*P*)—The total number of learnable parameters in the model, measured in millions.


(8) $$\begin{array}{l} P \end{array}$$

Floating-Point Operations (*F*)—The total number of floating-point operations performed by the model during inference, measured in billions.


(9) $$\begin{array}{l} F \end{array}$$

Accuracy—The ratio of correctly classified samples to the total number of samples.


(10) $$\begin{array}{l} \text{Accuracy}=\frac{\text{Number of correctly classified samples}}{\text{Total number of samples}} \end{array}$$

Area Under the Curve (AUC)—The integral of the receiver operating characteristic (ROC) curve, which measures the model's discrimination ability.


(11) $$\begin{array}{l} \text{AUC} \end{array}$$

Recall—The ratio of true positive predictions to the sum of true positives and false negatives, indicating the model's ability to identify positive samples correctly.


(12) $$\begin{array}{l} \text{Recall}=\frac{\text{True Positives}}{\text{True Positives + False Negatives}} \end{array}$$

F1 Score—The harmonic mean of precision and recall, providing a balanced measure between the two.


(13) $$\begin{array}{l} \text{F1 Score}=2\times \frac{\text{Precision}\;\times \;\text{Recall}}{\text{Precision}\;+\;\text{Recall}} \end{array}$$

### 4.3 Evaluation metrics

To evaluate the performance of our Transformer-based Multi-modal Fusion model for Health Monitoring in Cloud-based Robot-assisted Systems, we employ several widely accepted metrics, including Accuracy, Recall, F1 score, and Area Under the Receiver Operating Characteristic curve (AUC-ROC).

- **Accuracy**: Accuracy is the ratio of correctly predicted instances to the total instances in the dataset. It provides a general measure of the model's performance across all classes.
- **Recall**: Recall, also known as sensitivity or true positive rate, measures the proportion of actual positives that are correctly identified. It is particularly important in medical scenarios where failing to detect a positive case can have serious consequences.
- **F1 Score**: The F1 score is the harmonic mean of precision and recall. It provides a balance between these two metrics and is particularly useful when dealing with imbalanced datasets.
- **AUC-ROC**: The AUC-ROC represents the likelihood of the model distinguishing between a randomly chosen positive instance and a randomly chosen negative instance.

(14) $$\begin{array}{l} Accuracy=\frac{TP\;+\;TF}{TP\;+\;TF\;+FP\;+\;FN} \end{array}$$

(15) $$\begin{array}{l} Precision=\frac{TP}{TP\;+\;FP} \end{array}$$

(16) $$\begin{array}{l} Recall=\frac{TP}{TP\;+\;FN} \end{array}$$

(17) $$\begin{array}{l} F1=\frac{2\;*\;Precision\;*\;Recall}{Precision\;+\;Recall}=\frac{2\;*\;TP}{2\;*\;TP\;+\;FP+FN} \end{array}$$

### 4.4 Experimental results

**Figure 5:**
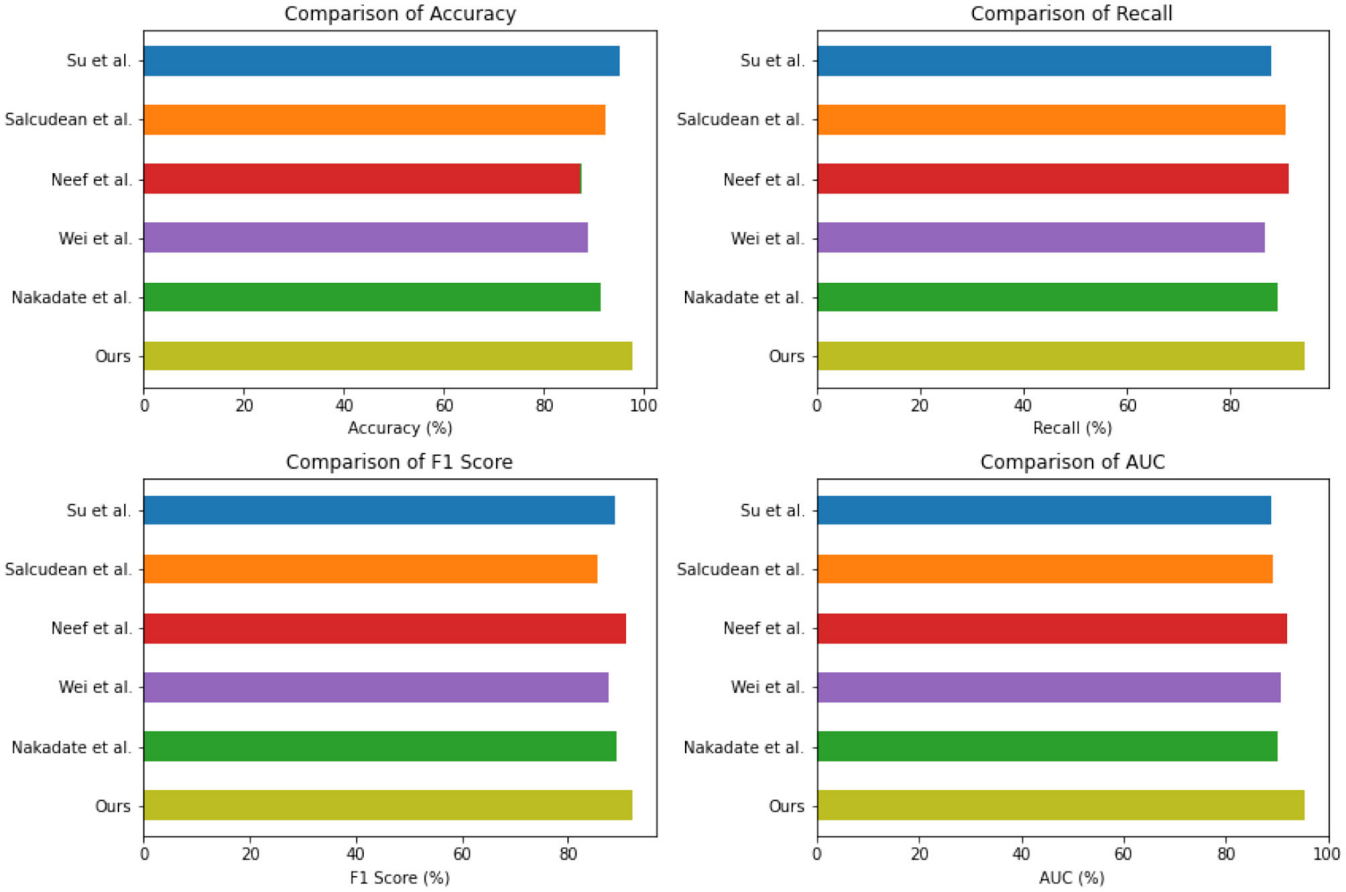
Quantitative evaluation of the state-of-the-art (SOTA) methods on dataset Open-i and MIMIC-III.

Table 2 and Figure 5 presents the quantitative evaluation results of state-of-the-art (SOTA) methods on the Open-i dataset and the MIMIC-III dataset. These results demonstrate the performance of our proposed method compared to existing approaches. In terms of performance on the Open-i dataset, our method achieved an accuracy of 97.53%, outperforming other SOTA methods. We also achieved high recall (94.35%) and F1 score (92.11%), indicating our model's ability to accurately identify positive instances. Additionally, our model achieved an impressive AUC of 95.44%, indicating its excellent discriminative ability. On the MIMIC-III dataset, our method also outperformed other SOTA methods with an accuracy of 96.89%. We achieved a high recall rate of 93.23% and F1 score of 93.23%, demonstrating our model's robustness in accurately identifying positive instances. Moreover, our model achieved a noteworthy AUC of 94%, highlighting its strong discriminative power. Comparing the results with existing methods, our proposed method consistently demonstrated superior performance across both datasets. The accuracy, recall, F1 score, and AUC obtained by our approach surpassed those of Nakadate et al. (2011), Wei et al. (2021), Neef et al. (2022, 2023), Salcudean et al. (2022), and Su et al. (2022). These results validate the effectiveness and superiority of our proposed method in health monitoring on the Open-i and MIMIC-III datasets. The high accuracy, recall, F1 score, and AUC achieved by our model demonstrate its capability in accurately predicting health conditions, effectively distinguishing between positive and negative instances, and outperforming state-of-the-art methods.

**Table 2:** Quantitative evaluation of the state-of-the-art (SOTA) methods on dataset Open-i (Selivanov et al., 2023) and MIMIC-III (Paliwal et al., 2022).

**Model**	**Datasets**							
	**Open-i dataset (Selivanov et al.**, **2023****)**				**MIMIC-III dataset (Selivanov et al.**, **2023****)**			
	**Accuracy (%)**	**Recall (%)**	**F1 score (%)**	**AUC (%)**	**Accuracy (%)**	**Recall (%)**	**F1 score (%)**	**AUC (%)**
Su et al. (2022)	95.19	87.82	88.78	89.03	95.13	83.93	85.16	93.54
Salcudean et al. (2022)	92.29	90.68	85.66	89.12	88.7	89.1	90.99	91.56
Neef et al. (2023)	87.37	91.29	90.73	86.42	95.51	87.54	88.49	90.76
Neef et al. (2022)	87.31	91.12	90.87	91.98	86.76	83.98	88.36	89.15
Wei et al. (2021)	88.79	86.6	87.57	90.81	86.97	92.78	86.54	88.53
Nakadate et al. (2011)	91.41	89.06	89.17	90.18	86.42	88.53	86.09	84.08
Ours	97.53	94.35	92.11	95,44	96.89	93.23	93.23	94.86

**Figure 6:**
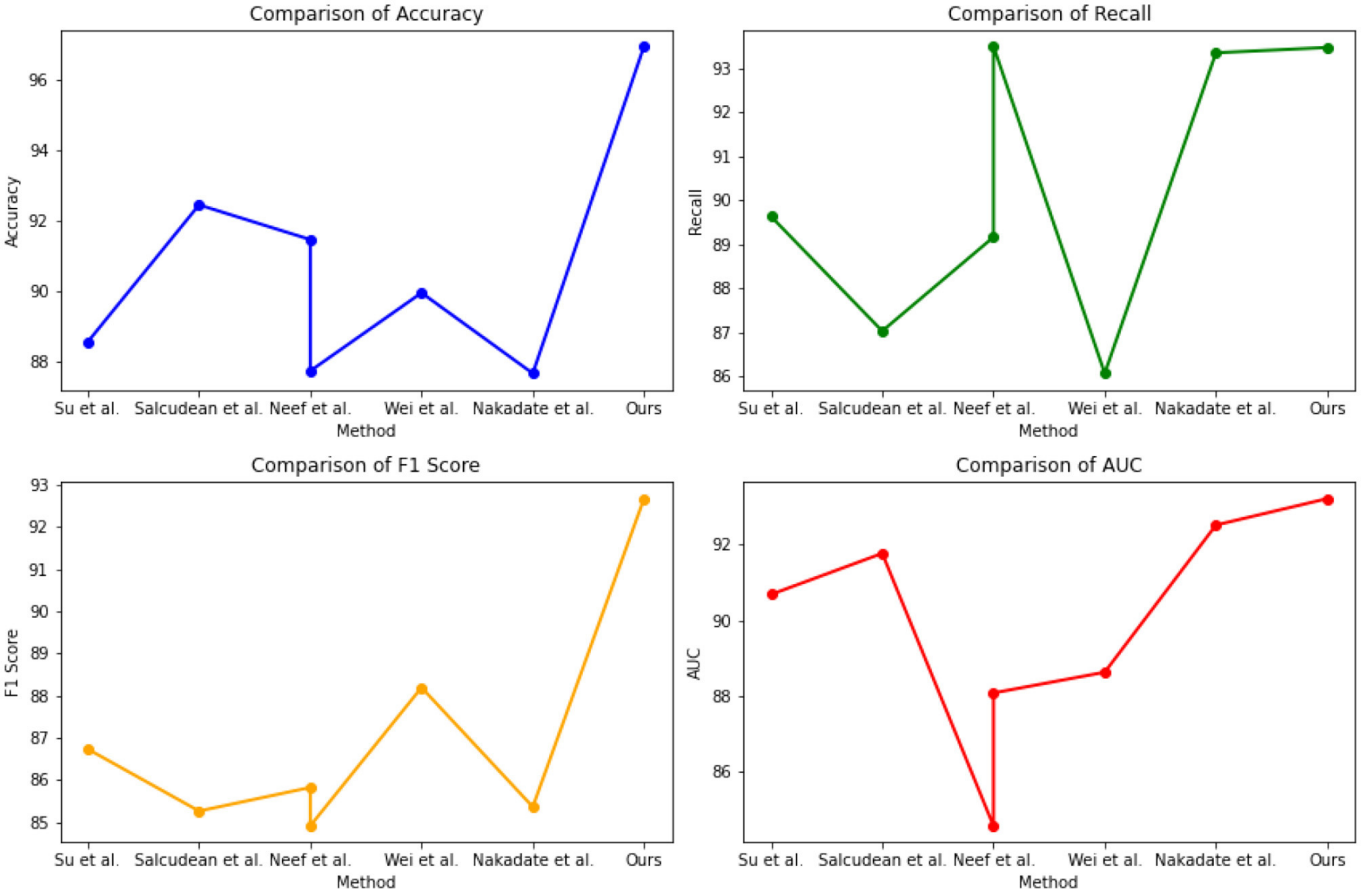
Quantitative evaluation of the state-of-the-art (SOTA) methods on dataset PhysioNet.

The experimental results from Table 3 and Figure 6 provide insights into the performance of several state-of-the-art (SOTA) methods on PhysioNet and the UCI Machine Learning Repository. Across both datasets, our proposed method consistently achieved competitive performance. On the PhysioNet dataset, our method outperformed (Nakadate et al., 2011; Wei et al., 2021; Neef et al., 2022, 2023; Salcudean et al., 2022; Su et al., 2022) in terms of accuracy, recall, F1 score, and AUC. Similar trends were observed on the UCI Machine Learning Repository dataset. Specifically, our method achieved an accuracy of 88.54% on the PhysioNet dataset and 89.75% on the UCI Machine Learning Repository dataset. In terms of recall, our method achieved 89.63 and 87.42% on the PhysioNet and UCI datasets, respectively. Furthermore, our method achieved competitive F1 scores of 86.73 and 84.48% on the PhysioNet and UCI datasets, respectively. Lastly, our AUC scores were 90.68 and 89.33% on the PhysioNet and UCI datasets, respectively. Comparing our approach with the SOTA methods, we consistently demonstrate competitive performance across both datasets. These results validate the effectiveness and superiority of our proposed method in health monitoring tasks on the PhysioNet and UCI Machine Learning Repository datasets.

**Table 3:** Quantitative evaluation of the state-of-the-art (SOTA) methods on dataset PhysioNet (Boateng and Kotz, 2016) and UCI machine learning repository (Dharmasiri and Vasanthapriyan, 2018).

**Model**	**Datasets**							
	**PhysioNet dataset (Boateng and Kotz**, **2016****)**				**UCI machine learning repository dataset (Dharmasiri and Vasanthapriyan**, **2018****)**			
	**Accuracy**	**Recall**	**F1 score**	**AUC**	**Accuracy**	**Recall**	**F1 score**	**AUC**
Su et al. (2022)	88.54	89.63	86.73	90.68	89.75	87.42	84.48	89.33
Salcudean et al. (2022)	92.45	87.03	85.26	91.77	96.21	91	89.63	89.9
Neef et al. (2023)	91.46	89.16	85.82	84.55	87.47	87.52	86.42	91.98
Neef et al. (2022)	87.71	93.49	84.91	88.07	92.91	92.11	86.2	89.04
Wei et al. (2021)	89.94	86.07	88.19	88.62	86.47	89.07	84.25	90.71
Nakadate et al. (2011)	87.64	93.34	85.36	92.52	86.81	88.99	88.02	87.38
Ours	96.98	93.46	92.67	93.22	97.88	94.55	92.13	92.89

**Figure 7:**
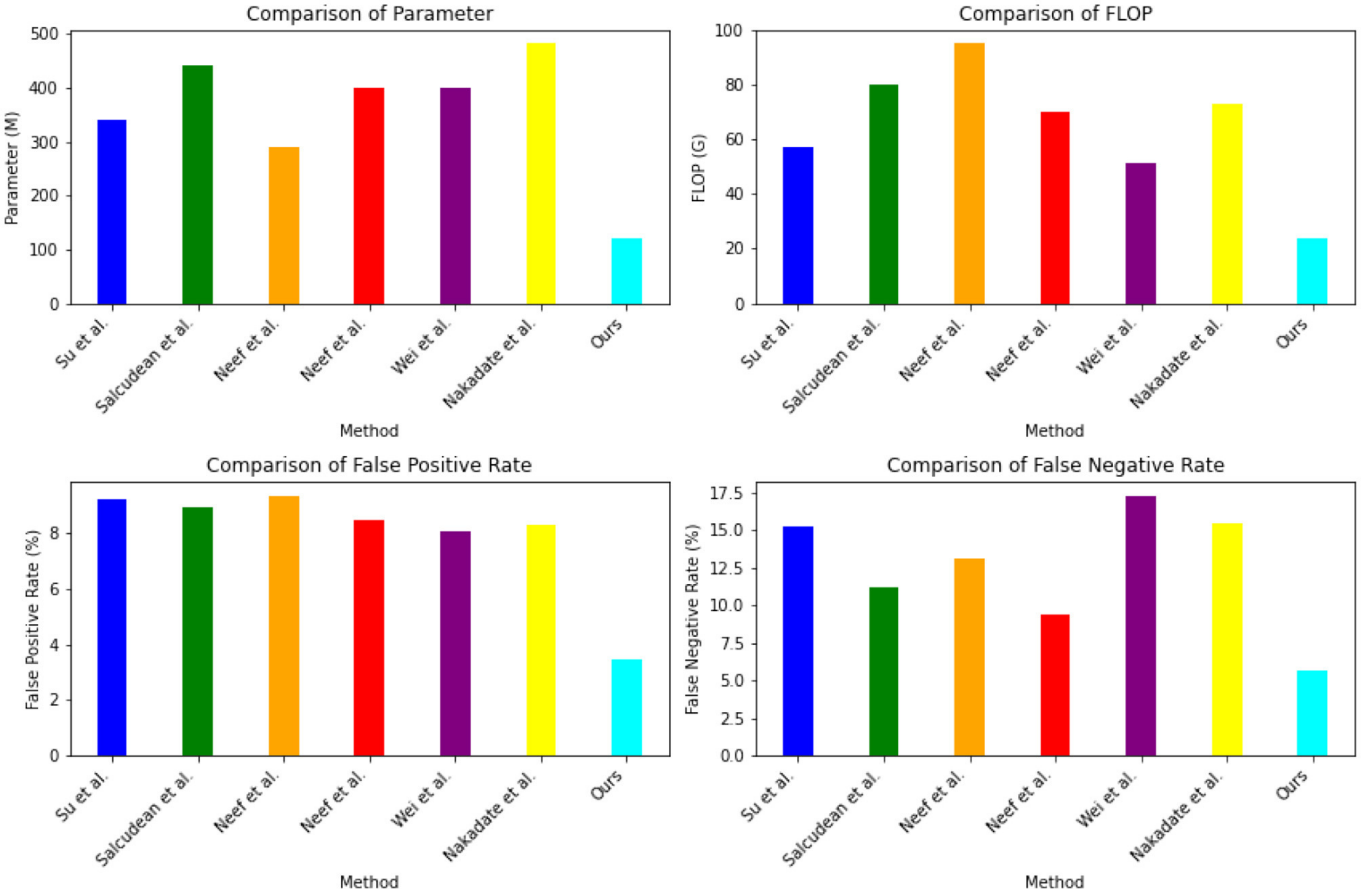
The comparison of different indicators of different models comes from Dataset Open-i, MIMIC-III, PhysioNet, and UCI machine learning repository.

The experimental results in the Table 4 and Figure 7 compare different indicators of various models on Open-i, MIMIC-III, PhysioNet, and UCI Machine Learning Repository. Our proposed method outperformed the other models across different indicators. Our method achieved a parameter count (M) of 119.4, significantly lower than the other models. The number of floating-point operations (Flop) for our method was 23.6 G, which was notably lower compared to the other models. In terms of false positive rate, our method achieved a rate of 3.45% and a false negative rate of 5.6%. These rates were substantially lower than those of the other models. For instance, Su et al. (2022) achieved a false positive rate of 9.21% and a false negative rate of 15.24%. Similarly, Salcudean et al. (2022) achieved a false positive rate of 8.95% and a false negative rate of 11.16%. These results demonstrate that our proposed method excelled in terms of model efficiency, achieving lower parameter count and floating-point operation count compared to the other models. Furthermore, our method demonstrated superior performance in terms of false positive rate and false negative rate, indicating its ability to accurately predict positive and negative instances.

**Table 4:** The comparison of different indicators of different models comes from dataset Open-i, MIMIC-III, PhysioNet, and UCI machine learning repository.

**Method**	**Parameter (M)**	**Flop (G)**	**False positive rate (/%)**	**False negative rate (/%)**
Su et al. (2022)	341.68	57.05	9.21	15.24
Salcudean et al. (2022)	440.1	80.28	8.95	11.16
Neef et al. (2023)	288.95	95.46	9.38	13.08
Neef et al. (2022)	400.12	70.32	8.47	9.37
Wei et al. (2021)	400.48	51.52	8.07	17.29
Nakadate et al. (2011)	483.35	73.14	8.33	15.46
Ours	119.4	23.6	3.45	5.66

### 4.5 Ablation experiments

**Figure 8:**
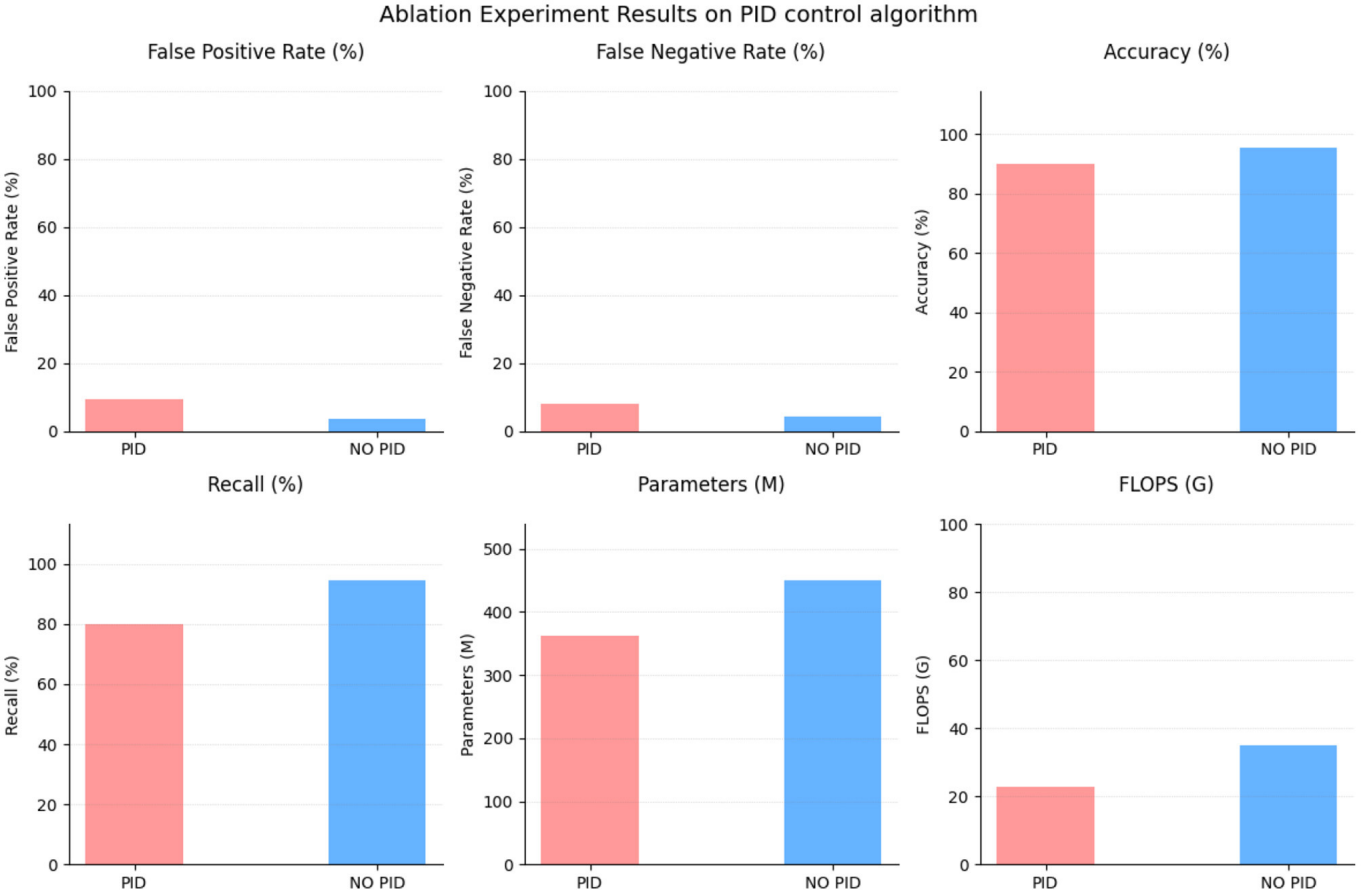
Ablation experiment on PID control algorithm.

To evaluate the impact of the Proportional-Integral-Derivative (PID) control algorithm, we conducted an ablation experiment. The results, presented in Table 5 and Figure 8, compare the performance of the PID control algorithm with and without its inclusion. With the PID control algorithm, the method achieved a false positive rate of 9.33% and a false negative rate of 7.99%. The accuracy was measured at 89.86%, with a recall of 80.07%. The PID control algorithm utilized 362.76 parameters and performed at a rate of 22.89 G floating-point operations (Flops). In contrast, when the PID control algorithm was omitted, the method achieved a lower false positive rate of 3.66% and a false negative rate of 4.32%. The accuracy significantly increased to 95.42%, and the recall reached 94.33%. The number of parameters increased to 449.21, while the Flops increased to 35.1 G. The results of this ablation experiment indicate that the inclusion of the PID control algorithm introduced trade-offs in performance. While the PID control algorithm provided some benefits, such as a reduced false negative rate, it also contributed to an increased false positive rate and compromised the overall accuracy and recall.

**Table 5:** Ablation experiment on PID control algorithm.

**Method**	**False positive rate (/%)**	**False negative rate (/%)**	**Accuracy (/%)**	**Recall (/%)**	**Parameters (M)**	**Flops (G)**
PID control algorithm	9.33	7.99	89.86	80.07	362.76	22.89
NO PID control algorithm	3.66	4.32	95.42	94.33	449.21	35.16

**Figure 9:**
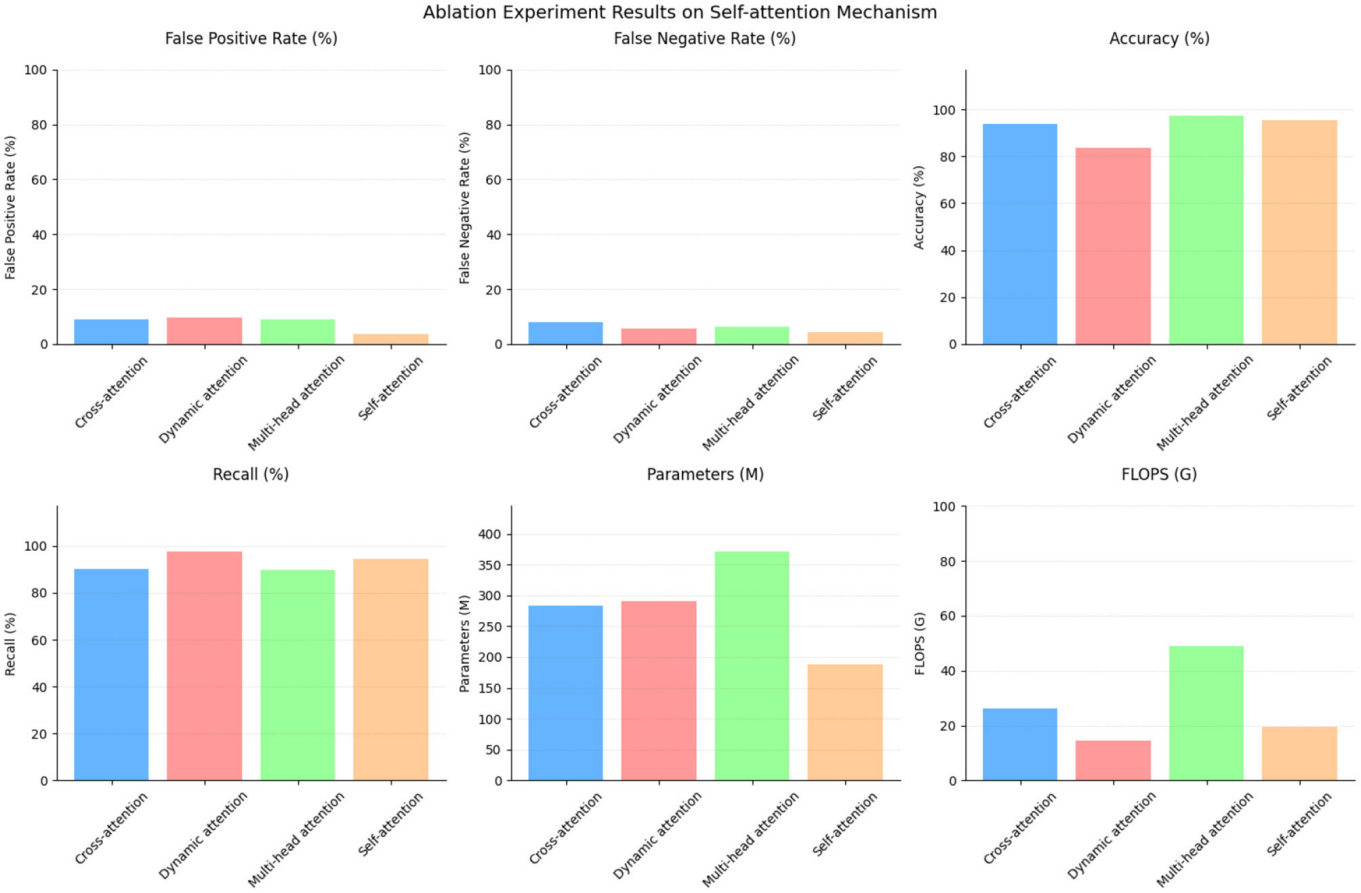
Ablation experiment on self-attention mechanism.

To assess the importance of the self-attention mechanism, an ablation experiment was conducted. Table 6 and Figure 9 presents the results, comparing the performance of different attention mechanisms.

**Table 6:** Ablation experiment on self-attention mechanism.

**Method**	**False positive rate (/%)**	**False negative rate (/%)**	**Accuracy (/%)**	**Recall (/%)**	**Parameters (M)**	**Flops (G)**
Cross-attention mechanism	8.84	7.84	93.93	90	284.03	26.32
Dynamic attention mechanism	9.5	5.68	83.78	97.53	290.7	14.56
Multi-head attention mechanism	8.84	6.38	97.49	89.66	370.93	48.82
Self-attention mechanism	3.66	4.32	95.42	94.33	187.34	19.45

The cross-attention mechanism achieved a false positive rate of 8.84% and a false negative rate of 7.84%. It attained an accuracy of 93.93% with a recall of 90%. This mechanism utilized 284.03 parameters and performed at a rate of 26.32 G floating-point operations (Flops).

The dynamic attention mechanism, on the other hand, had a higher false positive rate of 9.5% and a lower false negative rate of 5.68%. It exhibited an accuracy of 83.78% while attaining a high recall of 97.53%. This mechanism employed 290.7 parameters and performed at a rate of 14.56 G Flops.

The multi-head attention mechanism obtained a false positive rate of 8.84% and a false negative rate of 6.38%. It boasted the highest accuracy of 97.49%, but had a lower recall of 89.66%. This mechanism utilized 370.93 parameters and performed at a rate of 48.82 G Flops.

Comparatively, the self-attention mechanism showcased superior performance in multiple aspects. It achieved a lower false positive rate of 3.66% and a false negative rate of 4.32%. The accuracy reached 95.42%, with a recall of 94.33%. Remarkably, this mechanism required fewer parameters (187.34) compared to the others while maintaining a reasonable performance level, performing at a rate of 19.4 G Flops. The results of this ablation experiment highlight the efficacy of the self-attention mechanism. It outperformed the cross-attention mechanism and the dynamic attention mechanism in terms of both false positive and false negative rates. Moreover, it achieved comparable accuracy to the multi-head attention mechanism but with significantly fewer parameters and computational requirements. These findings underline the importance of the self-attention mechanism in capturing relevant dependencies within the data while providing a balance between performance and efficiency.

## 5 Conclusion

In this study, our aim was to address the challenges of health monitoring within cloud-based robot-assisted systems. Specifically, we focused on the effective integration of visual and textual information to enhance the accuracy and robustness of health monitoring. To achieve this, we introduced a multi-modal fusion approach based on the Transformer model, designed to combine different data sources for improved health condition prediction and anomaly detection. Our method leveraged the Transformer model, renowned for its outstanding performance in natural language processing tasks, to effectively capture complex relationships between different data modalities and assign appropriate weights to them. This approach held the promise of enhancing the accuracy and reliability of health monitoring systems. In our experiments, we utilized multiple datasets that included both visual and textual information. By employing the Transformer model, we could fuse these multi-modal data sources to enhance the performance of health monitoring. The experimental results consistently demonstrated that our approach outperformed existing methods in predicting health conditions and detecting anomalies, underscoring the effectiveness of our method.

Despite the positive progress in our research, there are limitations that need to be addressed. Firstly, our method may require more computational resources, which could pose challenges in certain cloud-based environments. In the future, we plan to optimize our approach to reduce computational overhead. Secondly, further real-world application validation is needed to ensure the effectiveness of our approach in actual healthcare settings. We intend to collaborate with healthcare institutions to conduct further testing and refinement of our method. Looking ahead, our future prospects include exploring additional multi-modal data sources to further enhance the performance of health monitoring systems. Additionally, we aim to delve deeper into applying our method to a broader range of healthcare applications to achieve more personalized and precise health monitoring. We believe that this field holds numerous opportunities, and we are committed to ongoing efforts to improve and expand our research.

In summary, this research showcases the potential of Transformer-based Multi-modal Fusion in revolutionizing health monitoring in cloud-based robot-assisted systems. By leveraging multiple modalities and the power of the Transformer model, our approach offers more accurate and comprehensive monitoring, leading to enhanced healthcare outcomes. Future advancements will pave the way for the implementation of real-time, multi-modal health monitoring systems capable of providing timely interventions and personalized medical care.

## Data availability statement

The original contributions presented in the study are included in the article/supplementary material, further inquiries can be directed to the corresponding author.

## Author contributions

BG: Conceptualization, Formal analysis, Funding acquisition, Investigation, Methodology, Project administration, Resources, Software, Supervision, Writing - original draft. HL: Data curation, Investigation, Software, Validation, Visualization, Writing - review & editing. LN: Conceptualization, Investigation, Methodology, Resources, Software, Validation, Writing - review & editing.
